# Leukocyte Count Restoration Under Dabrafenib Treatment in a Melanoma Patient With Vemurafenib-Induced Leukopenia: Case Report

**DOI:** 10.1097/MD.0000000000000161

**Published:** 2014-12-02

**Authors:** Elias Orouji, Birgit Ziegler, Viktor Umansky, Christoffer Gebhardt, Jochen Utikal

**Affiliations:** From the Skin Cancer Unit (EO, VU, CG, JU), German Cancer Research Center (DKFZ), Heidelberg; and Department of Dermatology, Venereology and Allergology (EO, BZ, VU, CG, JU), University Medical Center Mannheim, Ruprecht-Karl University of Heidelberg, Mannheim, Germany.

## Abstract

Recent advances in melanoma therapy have influenced the management of metastatic patients. Inhibitors of the BRAF/MEK/ERK signaling cascade have been proven highly effective in the metastatic disease although displaying different side effects.

Here, we report a patient with BRAF V600E-mutated stage IV melanoma who developed a severe leukopenia upon targeted therapy with the BRAF inhibitor vemurafenib. Interestingly, the immediate therapeutic switch to a different BRAF inhibitor ‘dabrafenib́ had no negative influence on the leukocyte count.

This case supports recent studies, which showed a differential influence of different BRAF inhibitors on patients’ leukocytes despite similar clinical efficacy in melanoma.

## INTRODUCTION

Targeted treatments of BRAF gene-mutated melanoma with BRAF (vemurafenib, dabrafenib) and MEK inhibitors have prolonged progression-free and overall survival.^1–3^ Interestingly, side effects differ among these targeted therapies. Dabrafenib treatment shows pyrexia in approximately 25% of patients,^1^ vemurafenib has no such effect.^3^ However, neutropenia was reported as an adverse event in some cases of vemurafenib treatment.^3^ Nevertheless, vemurafenib and dabrafenib have comparable clinical efficacy. Moreover, vemurafenib, but not dabrafenib, decreases patients’ peripheral lymphocyte counts and alters CD4^+^ T-cell phenotype and functions.^4^ Recently, Hong et al^5^ carried out an analysis of peripheral blood monocuclear cells obtained from patients treated with dabrafenib and found no changes in the absolute numbers of different lymphocyte subsets (T, B, and NK cells).

**FIGURE 1:**
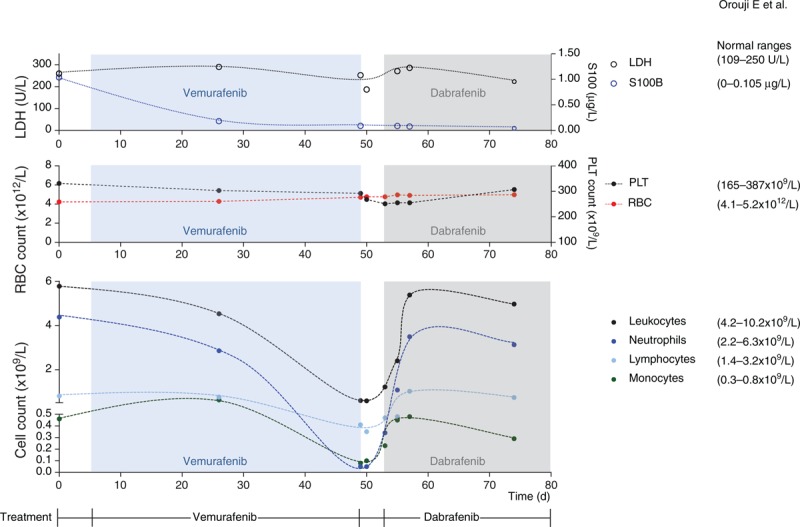
Changes in numbers of various leukocyte subsets as well as of LDH and S100B levels during targeted therapy with vemurafenib and dabrafenib. LDH = lactate dehydrogenase, PLT = platelets.

Here, we report a female white patient (64 years old) who was diagnosed with metastatic melanoma to multiple distant organ sites including brain, lung, liver, and kidney in November 2013. Because of brain metastases, a whole brain radiation therapy was started and a mutational analysis was performed revealing a BRAFV600E mutation. Therefore, targeted therapy with vemurafenib (960 mg orally twice daily) was started and the patient's condition became alleviated. However, the patient developed severe leukopenia (0.59 × 10^9^/L) and neutropenia (0.05 × 10^9^/L) (grade III based on Common Terminology Criteria for Adverse Events version 4.0) 5 weeks after the start of vemurafenib therapy. Numbers of other blood cell populations including red blood cells and platelets were within the normal ranges and did not undergo significant changes (Figure 1).

The brain metastases made withdrawal from targeted therapy problematic. Owing to recent studies, which showed that vemurafenib and dabrafenib have a differential influence on patients’ lymphocyte subsets despite similar clinical efficacy in melanoma,^4^ an immediate treatment with dabrafenib (150 mg orally twice daily) was started. A daily observation of leukocyte counts was performed. Interestingly, an increase in numbers of lymphocytes, neutrophils, and total leukocytes was observed under dabrafenib therapy, and this increasing trend continued over the next 4 days until the complete resolve of the leukopenia/neutropenia (Figure 1). However, during the leukopenia phase the patient did not receive granulocyte colony-stimulating factor.

A reduction of peripheral lymphocyte counts was previously related to melanoma progression rather than to its treatment.^6^ However, in our case, there was no progression of the disease during or after vemurafenib treatment; S100B levels were also decreasing upon the targeted therapy. Our case supports a recent publication showing a differential influence of targeted melanoma therapies on lymphocyte numbers.^4^ Inhibitors of the BRAF/MEK/ERK signaling cascade need also to be further assessed for immunomodulatory effects, in particular, when applied in planned combination therapies with other agents such as inhibitors of negative immune checkpoints (eg, anti-CTLA4 or anti PD-1/PD-L1 antibodies).
